# Case Report: Pitfalls in bone marrow evaluation: importance of adequate bone marrow sampling

**DOI:** 10.3389/fonc.2025.1628130

**Published:** 2025-11-20

**Authors:** Alireza Ghezavati, Elham Vali Betts, Ananya Datta Mitra

**Affiliations:** Department of Pathology and Laboratory Medicine, University of California Davis, Sacramento, CA, United States

**Keywords:** bone marrow, lymphoma, pitfall, mantle cell lymphoma, lymphoplasmacytic lymphoma, classic Hodgkin lymphoma

## Abstract

Bone marrow evaluation is a powerful diagnostic tool, but it comes with several potential pitfalls. These include issues related to sampling errors, technical challenges during processing, and misinterpretation of the findings due to similarities between various diseases. Awareness of these pitfalls, adopting a systematic approach of reviewing the bone marrow samples, and carefully integrating clinical information are critical to ensuring accurate diagnosis. Using ancillary techniques, such as immunohistochemistry (IHC), can further aid in distinguishing between benign reactive changes and malignant processes, reducing the likelihood of diagnostic errors. Bone marrow sampling is inherently challenging, and improper or inadequate sampling is one of the most common reasons for diagnostic failure. Moreover, the process of collecting and preparing the bone marrow samples, leading to aspicular aspirate smears with hemodilution, or tissue preparation techniques like decalcification procedures in the core biopsy, leading to IHC stain failures, may add to the diagnostic challenges in bone marrow evaluation. Lastly, inherent properties of some diseases or the presentation of abnormal findings with focal involvement or obscuring of morphology in an inflammatory background can pose a potential diagnostic challenge. In this article, we present three diagnostically challenging cases that highlight potential pitfalls in bone marrow evaluation, along with a brief review of the literature, and describe strategies to avoid diagnostic errors based on our institutional experience.

## Introduction

Bone marrow assessment is an integral part of hematopathology evaluation because it not only provides a comprehensive understanding of the state of different cellular elements of the marrow but also helps to diagnose a wide range of hematological and non-hematological (solid-organ metastases, metabolic diseases, and infections) (1) conditions, guide therapy, and offer prognostic information. By examining bone marrow directly, pathologists can identify abnormalities that may not be evident from peripheral blood alone, offering critical insight into the patient’s condition and enabling more accurate diagnosis and treatment strategies. Furthermore, the evaluation of the marrow can also help in following the patients after chemotherapy and bone marrow transplant (2). Indications for bone marrow biopsy include unexplained cytopenias (e.g., hemoglobin <10 g per deciliter, absolute neutrophil count <1.5 × 10^9^ per liter, or platelet count <100 × 10^9^ per liter), unexplained cytosis (e.g., leukocytosis or thrombocytosis), the presence of atypical or immature cells on peripheral blood smear, lymphadenopathy or splenomegaly of uncertain etiology, abnormal marrow signals on imaging studies, staging or follow-up of hematologic malignancies, and evaluation of potential stem cell donors (3). The evaluation of the bone marrow encompasses cytomorphological examination of the bone marrow aspirates, touch preps, clot section and trephine core biopsy with additional tests sent for flow cytometry and immunohistochemistry, fluorescence *in situ* hybridization (FISH), molecular studies, chimerism, and cytogenetic analyses (4).

Bone marrow is very heterogeneous, and adequate bone marrow evaluation depends on the methods of sample collection, preparation, processing, and reporting of bone marrow. Inconsistencies in any of these factors may result in discrepancies in diagnosis or classification that ultimately will affect treatment and clinical outcomes (5). Some of the most common causes leading to pitfalls in hematopathology include lack of adequate material on the slide, like a “dry tap” on aspirate smears or extremely subcortical core biopsy; lack of proper history and clinical or radiological workup; and finally even with sufficient clinical background and adequate material, an unsatisfactory workup can lead to a potential diagnostic error. In the literature, the diagnostic sensitivity of bone marrow aspiration and biopsy for hematologic disorders varies according to the underlying disease. Aspirate sensitivity may reach approximately 90% in acute leukemias and multiple myeloma, but it is substantially lower (65% or less) in focal infiltrative processes such as lymphoma (6–8). Reported rates of specimen inadequacy including hemodilution, dry tap, and insufficient core length range from 2% to 10%, depending on the criteria used and operator experience (9, 10). In our laboratory, bone marrow aspiration and biopsy are performed according to standard institutional protocols, consistent with international guidelines from the World Health Organization and the College of American Pathologists. Specimens are obtained from the posterior superior iliac crest under aseptic conditions using local anesthesia. Aspirate smears are prepared immediately for morphological evaluation, and core biopsy specimens are fixed in formalin and decalcified for histologic examination. Sample adequacy is defined by the presence of spicules in the aspirate smears and a core length of at least 1.5–2.0 cm containing evaluable marrow elements without extensive crush artifact or hemodilution.

Here, we will discuss the importance and challenges of bone marrow evaluation in hematopathology through various case presentations.

## Bone marrow evaluation criteria used for all cases

Bone marrow trephine biopsies were fixed in 10% neutral-buffered formalin, decalcified in phosphoric acid buffer, and embedded in paraffin. Sections (3–4 µm) were stained with hematoxylin and eosin (H&E) and Giemsa for morphological evaluation prior to immunohistochemical analysis. Reticulin staining (Gomori silver impregnation) was performed for the assessment of marrow fibrosis. Each biopsy specimen was required to contain a minimum of 10 evaluable intertrabecular marrow spaces to be considered adequate for interpretation.

### Case 1

The patient is an 84-year-old woman with a past medical history of ischemic stroke (diagnosed and treated in 2023) who presented to the emergency department (ED) with rectal bleeding, weakness, and vertigo. The complete blood count (CBC) revealed leukocytosis (16.1 K/mm^3^), mostly composed of atypical and mononuclear cells with blast-like morphology. The peripheral blood flow cytometry revealed two abnormal B-cell populations (47% in aggregate), both lambda-restricted. The predominant abnormal population is positive for CD10 and equivocal CD5; however, the second and minor population is CD10-negative with CD5 expression. Moreover, approximately 2% CD34-positive myeloblasts were noted with no immunophenotypic aberrancy. In addition, CBC revealed a microcytic anemia and thrombocytopenia.

**Figure 1:**
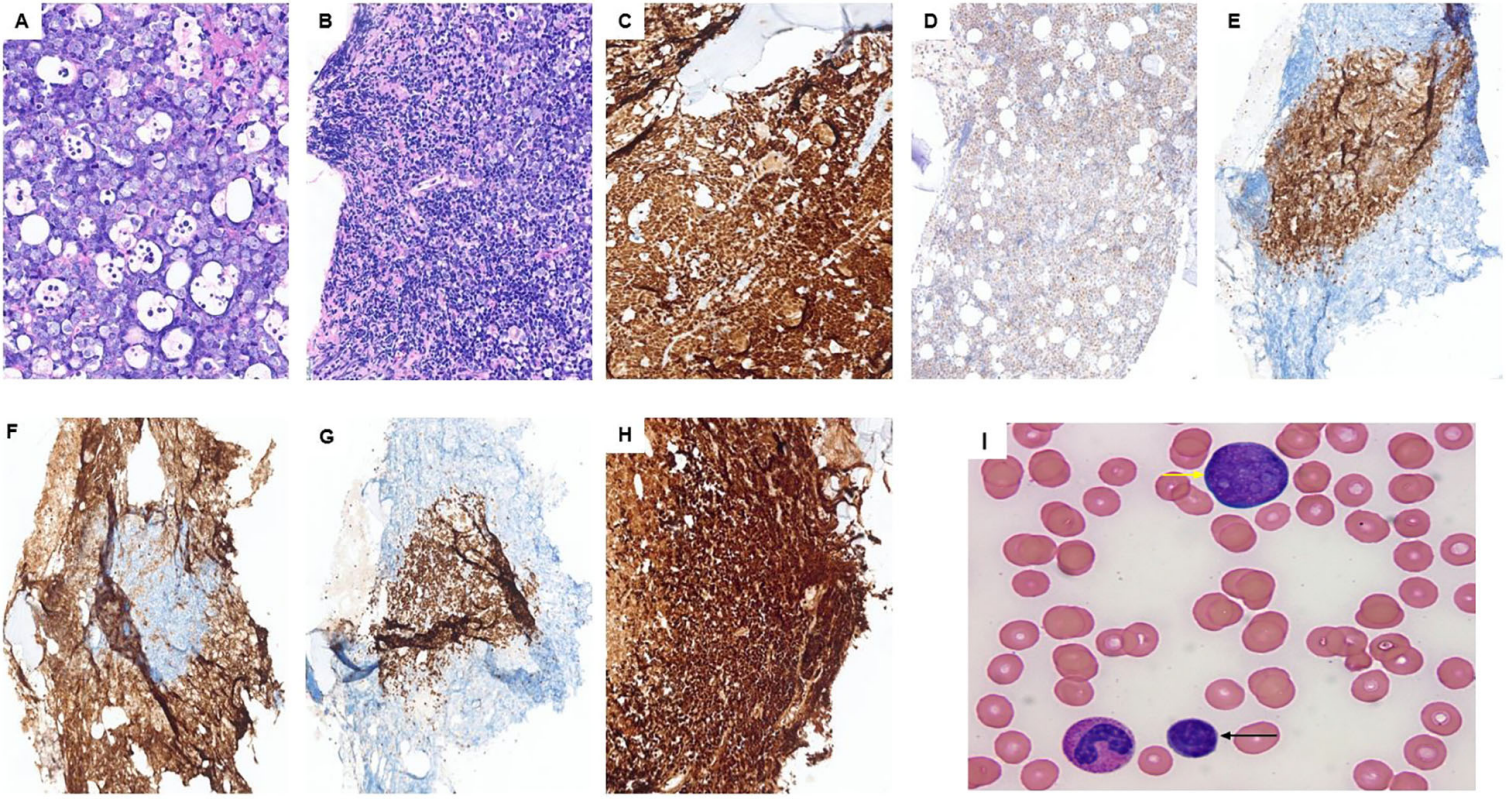
Case 1: **(A)** blastoid component; **(B)** mostly low-grade portion of the lymphoma although some blastoid component can be seen on the top and right side. Both classic and blastoid components express PAX5 **(C)**, SOX11 **(D)**, and P53 **(H)**. CD5 **(E)** and BCL-2 **(G)** highlight the low-grade portion of the lymphoma, while CD10 **(F)** highlights the high-grade subset surrounding the classic MCL. **(I)** Peripheral blood smear showing small lymphoma cells (black arrow) and the blast-like lymphoma cells with prominent nucleoli (yellow arrow) in the peripheral blood.

Subsequently, a bone marrow evaluation was performed with a core biopsy of at least 1.7 cm in length, revealing a hypercellular marrow for the age (~90%), extensively involved by sheets of medium- to large-sized cells with blastoid morphology (>90%) (**Figure 1A**). In addition, an area of atypical small lymphoid cells was identified (**Figure 1B**). Medium/large cells had a vesicular chromatin and one to multiple nucleoli with many apoptotic bodies and increased mitotic figures, while small cells had irregular nuclear contours with inconspicuous nucleoli. Both populations expressed B-cell markers (CD20 and PAX5) as well as cyclin D1 and SOX11. c-MYC and diffuse strong mutation-type P53 expression were only observed in the high-grade areas. P53 IHC could not be assessed in the low-grade component due to exhaustion of the tissue in the low-grade areas, and c-MYC was negative in the low-grade areas. Both populations were negative for CD23, CD34, and TdT. The medium/large cell component was positive for CD10 and negative for CD5 and BCL-2, while the small cell component was positive for CD5 and BCL-2 and negative for CD10 and c-MYC (**Figures 1C–H**). Ki67 showed an extremely high proliferation index of >95% in the medium- to large-sized cells with blastoid morphology.

A peripheral blood (PB) flow cytometry showed a lambda-restricted population of lymphoma cells accounting for ~47% of total PB cellularity. This population was composed of two subpopulations. The first one was CD5^−^/CD10^+^ and large-sized (based on the forward scatter), accounting for approximately 18% of lymphoma cells, and the second subpopulation was CD5^+^/CD10^−^ and small-sized (based on the forward scatter), accounting for approximately 82% of lymphoma cells. A peripheral smear was reviewed, showing the lymphoma cells (**Figure 1I**). The peripheral blood FISH study confirmed the presence of *CCND1:IGH* fusion as well as the *MYC* rearrangement. In addition, *TP53* mutation (*TP53* exon 6: c.614A>G (p.Y205C)) was identified by mutation analysis.

Overall, the findings were consistent with the diagnosis of mantle cell lymphoma (MCL), concomitant classic and blastoid subtypes. The findings supported that the blastoid component is transformed from the low-grade classic MCL. Interestingly, during the transformation, we see an immunophenotypic shift. In the transformation process in this case, lymphoma cells lost CD5 and BCL-2 and acquired CD10. In addition, we see multiple features associated with an adverse clinical course in this case, including diffuse P53 expression by IHC, *TP53* mutation, high Ki67 proliferation index, and transformed blastoid MCL (compared to *de novo*) (11–13). Unfortunately, this patient passed away 8 days after this bone marrow biopsy.

#### Diagnostic challenge in this case

Blastoid MCL can be found as a *de novo* lymphoma or rarely as a transformation from classic MCL (13). In our case, most of the lymphoma is the blastoid subtype with a small portion of classic MCL. However, probably if the evaluation and bone marrow biopsy are performed earlier in the course of the disease in this patient, we would see more low-grade component and less high-grade/blastoid subtype. Also, in this case, there is a chance of missing the high-grade component if we receive insufficient tissue, especially from a subcortical core biopsy or a lack of aspirate morphology. Therefore, it is crucial to have adequate sampling and enough tissue to rule out a high-grade component in cases of MCL, and if this requirement is not met, it should be mentioned in the pathology report to warrant clinicians about the possibility of a concurrent high-grade component, which may be missed.

### Case 2

**Figure 2:**
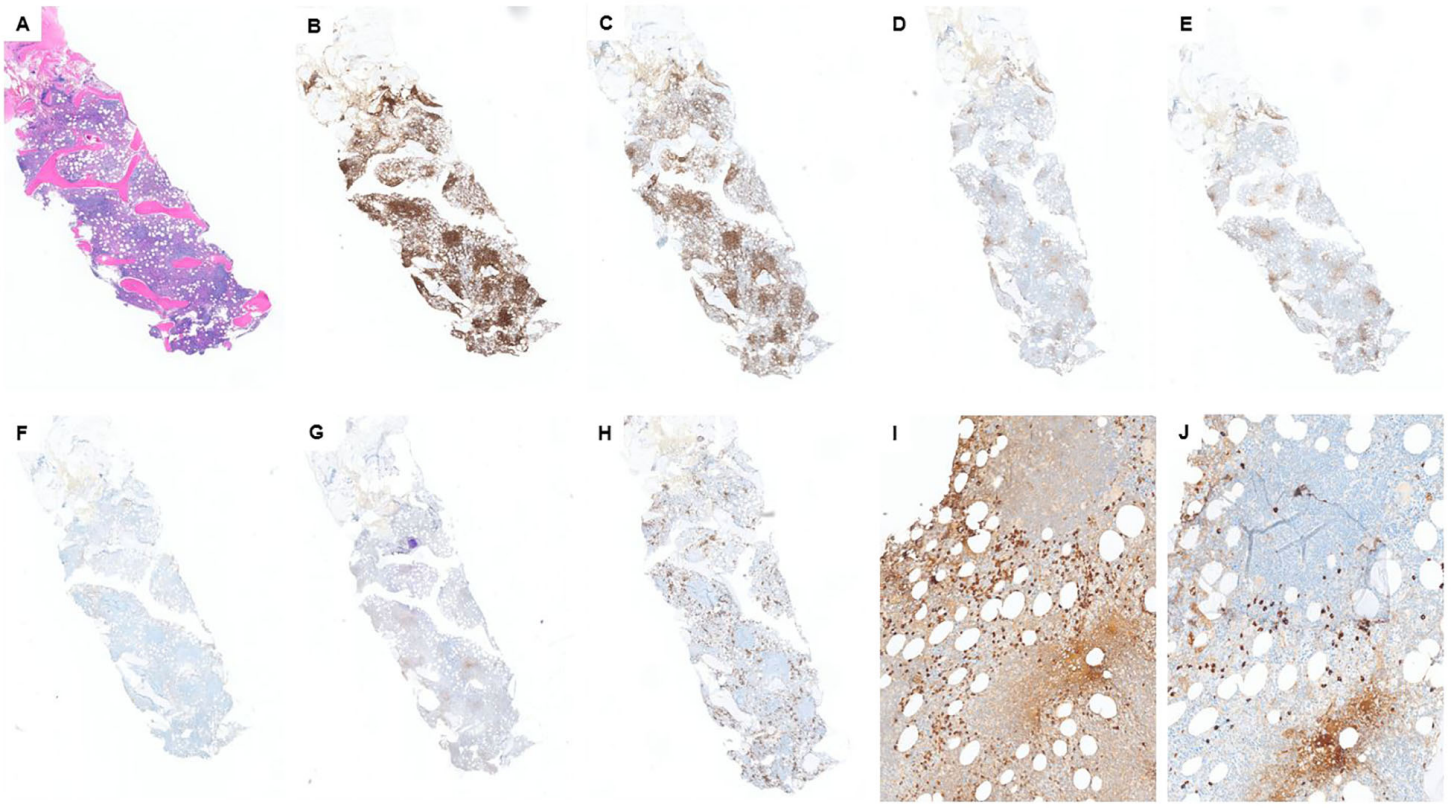
Case 2: **(A)** H&E shows a hypercellular marrow with multiple lymphoid aggregates. Lymphoid aggregates are mostly positive for CD20 **(B)** and PAX5 **(C)** and are negative for CD5 **(E)**, CD10 **(F)**, and LEF1 **(G)**. CD3 **(D)** highlights some T cells, scattered and in small aggregates. CD5 is similar to CD3. CD138 **(H)** highlights approximately 10% plasma cells, which are mostly positive for kappa by IHC **(I)**. However, we still see some lambda-positive plasma cells **(J)** with a kappa:lamda ratio of ~5:1.

The patient is a 69-year-old man with a reported history of possible chronic lymphocytic leukemia (CLL). Physical examination is notable for mild anterior mandibular lymphadenopathy. The PET/CT showed widespread lymphadenopathy, involving the neck, chest, abdomen, and pelvis. The patient underwent a bone marrow biopsy as a surveillance workup for “CLL.” The bone marrow biopsy showed extensive involvement by a CD5^−^/CD10^−^ low-grade B-cell lymphoma. Lymphoma cells were small in size (**Figure 2A**) with mature chromatin, and positive for CD19, CD20, PAX5, and BCL-2, and negative for CD5, CD10, CD23, cyclin D1, and LEF1 (by IHC and/or flow) (**Figures 2B–G**). CD138 IHC highlighted approximately 10%–12% plasma cell population with a major subset positive for kappa (kappa:lambda ratio of ~5:1) (**Figures 2H–J**). Concurrent flow cytometry showed kappa-restricted B cells and showed mildly kappa-predominant plasma cells (<0.5% of total events with a kappa:lambda ratio of ~4:1). The peripheral blood and CBC also showed absolute lymphocytosis (38.9 K/μL), mostly composed of atypical small lymphocytes. Our morphologic and immunophenotypic findings did not support the clinically suspected diagnosis of CLL.

Furthermore, 6 months of prior serum protein electrophoresis and immunofixation (SPEP/IFE) studies showed two different monoclonal paraproteins (0.4 g/dL in total) with concordant light chains and discordant heavy chains (IgG kappa and IgM kappa). The most recent SPEP showed an increased amount of M protein to 0.7 g/dL. In addition, serum kappa free light chain was significantly increased to 1,184.84 mg/L (normal range: 3.30–19.40 mg/L) with an increased kappa:lambda ratio of 33.26 (normal range of 0.26–1.65). Serum lambda free light chain was mildly increased to 35.62 mg/L (normal range of 5.71–26.30 mg/L).

The specimen was sent for molecular/FISH studies. *MYD88* mutation was detected. Plasma cell myeloma FISH studies detected *D13S319* deletion and *IGH* rearrangement. Moreover, plasma cell myeloma IgH complex FISH detected *CCND1:IGH* translocation; however, this genetic alteration was not detected in non-Hodgkin lymphoma FISH studies. We consulted the molecular lab, which verified the accuracy of both studies and confirmed that *CCND1:IGH* translocation was detected in the plasma cell-rich sample.

We can see multiple evidence favoring the diagnosis of lymphoplasmacytic lymphoma (LPL), including serum IgM kappa, the presence of kappa-restricted CD5^−^/CD10^−^ small B-cell lymphoma, and the detection of *MYD88* mutation (this mutation has not been reported in a plasma cell neoplasm). On the other hand, there are multiple lines of evidence of a concurrent plasma cell neoplasm (PCN), such as the presence of serum IgG kappa, kappa predominant plasma cells on the core biopsy, and detection of PCN-associated genetic alterations (*13q* deletion and *CCND1:IGH*). Overall, the case is signed out as “concomitant LPL and a plasma cell neoplasm.”

#### Diagnostic challenge in this case

PCN and LPL/Waldenström macroglobulinemia (WM) are two distinct B-cell lymphoproliferative neoplasms with different clinical courses and therapeutic approaches. Their co-existence is a rare condition and challenging to diagnose, as there is extensive laboratory/morphologic/immunophenotypic overlap between these two entities (14, 15). The reason for this rarity can be due to misclassification, as concurrent PCN may have been classified as the plasmacytoid component of the LPL (14). However, the coexistence of a PCN with other mature B-cell lymphomas, particularly CLL, is not rare (14, 16).

Some features have been described that can help us diagnose this co-existence. For example, LPL is usually associated with IgM monoclonal paraprotein and rarely produces IgA or IgG; however, PCNs often produce IgG or IgA monoclonal proteins (15, 17, 18). In addition, the presence of dual serum monoclonal paraproteins (particularly heavy chain isotype discordant) favors the diagnosis of concurrent LPL and a PCN (14). Wang et al. showed in their case series that in the majority of these cases, there is heavy chain discordance, while we have light chain concordance (19).

The other clue that can be helpful is molecular studies. *MYD88* mutation is a relatively sensitive and specific finding in LPL cases; however, this mutation is not found in PCNs, including IgM-producing myelomas, and it is not detected in many of the other low-grade B-cell lymphomas as well (15). On the other hand, *CCND1:IGH* translocation can be found in PCNs, but not in LPLs (15, 18). Therefore, the findings of concurrent *MYD88* mutation and genetic alterations, which are more common in PCNs, like *CCND1:IGH*, favor the diagnosis of concomitant LPL and a PCN. Wang et al. also introduced some other criteria that can be helpful in this situation. For example, the presence of a neoplastic plasma cell with immunophenotypic aberrancy (like CD56 and cyclin D1 expression or loss of CD19) in an LPL case suggests a concurrent PCN (19). Another differential diagnosis in this situation can be the lymphoplasmacytic variant of multiple myeloma. However, there is evidence that makes this differential diagnosis less likely. In the lymphoplasmacytic variant of MM, we should have one single neoplastic population with two distinct morphologic features, and it has been shown that these cells co-express both plasmacytic markers (like CD138) and B-lymphoid markers (like CD20 and PAX5) (20). However, in our case, we identified two distinct neoplastic components: one malignant B-cell component expressing B-cell markers and one malignant plasma cell component expressing plasma cell markers with no overlap between these two malignant populations (immunophenotypic characteristics confirmed by flow cytometry). The plasma cell neoplasm component of our case is best classified as smoldering myeloma, as we identified approximately 10%–12% clonal plasma cells in the marrow with no myeloma-defining event as defined by the CRAB criteria (hypercalcemia, renal insufficiency, anemia, and bone lesions). There were no hypercalcemia (serum calcium levels were approximately 8 mg/dL), no renal insufficiency (serum creatinine 1.54 mg/dL), no lytic lesions in the bone, and no anemia (Hb: 14 g/dL) in this patient. Serum creatinine has been increased constantly with the most recent value of 1.54 mg/dL, but it never reached the threshold of 2 mg/dL.

Thus, the co-existence of LPL and a PCN is a rare condition (likely due to misclassification), and we, as pathologists, need to be aware of this entity and do a diligent workup with serological, radiological, and clinical bone marrow evaluation and molecular studies in suspicious cases to make sure we are not misclassifying the PCN as a plasmacytoid component of LPL.

### Case 3

The patient was a 59-year-old man with past medical history of hypertension, hyperlipidemia, and diabetes, who presented with 4 months of 40-lb weight loss, fevers/chills, and night sweats and had been evaluated at outside hospitals. He presented to our ED with intractable vomiting, fatigue, and reduced appetite. Review of records showed pancytopenia (WBC: 3.3 K/mm^3^; HB: 10.6 g/dL; and platelet count: 74 K/mm^3^) with differential showing monocytosis (0.6 K/mm^3^), lymphopenia (0.1 K/mm^3^), elevated transaminase levels (AST/ALT: 60/47 U/L), mild porta hepatis lymphadenopathy (11 mm), and splenomegaly noted on CT abdomen pelvis. PET/CT showed similar retroperitoneal and porta hepatis lymphadenopathy with just below/equivalent (^18^fluoro-deoxyglucose) FDG uptake to the liver. Bone marrow was isodense to the liver on PET-CT. A bone marrow evaluation was done, and the aspirate smears were cellular, showing erythroid-predominant trilineal hematopoiesis with no overt dysplasia or increase in blasts. There were scattered hemophagocytic histiocytes noted in the aspirate material, including forms that had ingested one to few nucleated erythroid cells. The core biopsy was hypercellular, which showed multiple foci of non-necrotizing granulomatous inflammation containing numerous histiocytes, some lymphocytes and eosinophils, and scattered few large cells with vesicular nuclei, prominent nucleoli, and pale cytoplasm. No definite Reed–Sternberg-like cells were noted. Scattered few plasma cells were in the marrow space, not associated with the granulomas. Given the paucity of these large cells, interpretation of the IHC studies is difficult.

**Figure 3:**
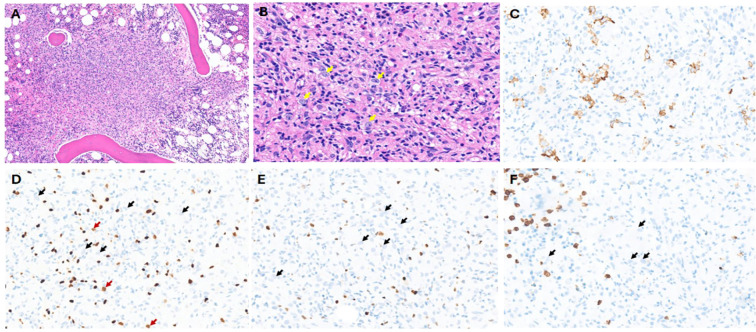
Case 3: **(A)** H&E showing lymphohistiocytic infiltrate with poorly formed granulomatous inflammation (×20 magnification). **(B)** Yellow arrows show very few scattered large cells with vesicular chromatin and prominent nucleoli (×40 magnification). No definite RS cells are noted. **(C)** CD30 immunohistochemistry (IHC) showing positive cells; however, it did not show the classic membranous and Golgi staining (×40 magnification). **(D)** PAX5 IHC: Red arrows show a subset of possible dim-positive cells, while black arrows show negative large cells. Background darkly stained small B cells are noted (×40 magnification). **(E)** MUM1 IHC: black arrows show negative large cells (×40 magnification). **(F)** CD15 IHC: Black arrows show negative large cells. Background granulocytes are highlighted (×40 magnification).

The large, atypical cells seen in the H&E were positive for CD30 but appeared negative largely for CD20, CD15, and MUM1 (**Figures 3A–F**). They were negative for EBV by EBER-*in situ* hybridization (ISH) studies. Pax 5 stains scattered small lymphocytes strongly, but dim PAX5 in the atypical foci was not observed, and there were few possible dim cells, but it is inconclusive (**Figure 3D**). In addition, the MUM1-positive cells were variable in size. The reticulin fibrosis was markedly increased in the granulomas. Concurrent flow cytometry studies showed no immunophenotypic abnormalities. Overall, the marrow biopsy study showed two distinct findings. The first is the presence of hemophagocytic histiocytes in the aspirate material, which, in correlation with clinical criteria, met the diagnosis of hemophagocytic lymphohistiocytosis (HLH). Additionally, the core biopsy shows atypical lymphohistiocytic aggregates with associated reticulin fibrosis. The morphologic appearance is suspicious for the possibility of marrow involvement by malignant lymphoma, such as classic Hodgkin lymphoma (CHL). However, definitive classification and diagnosis are challenging, especially considering the paucity of large, atypical cells and the limited ability to define a reliable phenotype for these cells. Furthermore, the lack of widespread lymphadenopathy in this case is challenging for a diagnosis of a lymphoma, as no tissue can be targeted for a reliable biopsy evaluation. The patient received a subsequent frontline chemotherapy regimen, ABVD (doxorubicin, bleomycin, vinblastine, and dacarbazine), and a subsequent bone marrow biopsy showed no evidence of residual disease. However, due to immunosuppression, the patient developed *Aspergillus* fungemia and succumbed to his infection.

#### Diagnostic challenge in this case

CHL is a mature B-cell lymphoma with the majority of cases predominantly affecting the lymph nodes and can extend to extranodal sites. Diagnosis of CHL is based on morphology and immunohistochemistry evaluation showing the characteristic Reed–Sternberg (RS) cells with owl’s eye nucleus and prominent nucleoli and showing positive staining for PAX5 (dim), CD30, CD15, and MUM1 and negativity for CD20, CD45, OCT2, and/or BOB1 (21). Diagnostic dilemma might result from the inconsistent morphologic features between the primary site of disease and the subsequently affected organs (22, 23). Moreover, limited foci of extranodal tissue involvement and scarcity of classic RS cells in a mixed inflammatory and fibrotic background may lead to potential pitfalls in diagnosis. Bone marrow (BM) involvement by CHL is rare and occurs in a small subset of patients at primary diagnosis. It is generally accompanied by diffuse lymphadenopathy and is associated with an advanced stage of disease (24, 25). Isolated BM involvement by CHL is very rare and only described in case reports (23, 26). The presence of RS cells in a suitable inflammatory background is essential for the diagnosis of CHL involving the marrow (27). However, this needs to be confirmed by the classic immunophenotype of the RS cells showing expression of CD15, CD30, and PAX5 (28–30). Thus, morphological pattern recognition of CHL involving the bone marrow is extremely important, followed by immunophenotypic confirmation.

Previous reports have shown that the majority of the cases of CHL involving the marrow presented with lymphadenopathy and hepatosplenomegaly and B symptoms, followed by CBC abnormalities with anemia being most frequently observed, followed by leukocytosis, thrombocytopenia, and thrombocytosis (31). Most of the cases of CHL involving the marrow were initially diagnosed in the lymph node, followed by marrow evaluation showing involvement. Only four cases had a primary bone marrow diagnosis and two cases with a subsequent confirmation on a nodal biopsy (31). The pattern of bone marrow involvement as described in this study (31) was extensive (>50% involvement) in the majority of the cases and showed almost equal prevalence of either fibrous or histiocyte-rich morphology. In all these cases, classic RS morphology was observed in the cells showing pale vesicular chromatin, owl’s eye nucleoli, and abundant eosinophilic cytoplasm. A few mummified variants and lacunar cells were also observed. In all these cases, the RS cells were positive for CD30 and PAX5 with subset expression of CD15 and EBER. A diffused increase in reticulin fibrosis was noted, and grade 3 myelofibrosis (MF) was most frequently observed followed by grade 2 and grade 1. In one report (32), cases with granulomatous inflammation in the marrow were misinterpreted, and a diagnosis of CHL was misinterpreted due to the obscuring of lymphoma cells by the granulomatous reaction.

Recent advancements in PET/CT have shown high concordance with pathological evaluation (including bone marrow biopsy) and have provided a highly sensitive and specific alternative non-invasive way of staging CHL (33, 34). While PET/CT has largely replaced BM biopsy for initial staging in classical Hodgkin lymphoma, BM biopsy is still recommended as a standard modality when there is concern about false-negative results from PET/CT. It provides a direct, histological confirmation of lymphoma involvement, which is critical for accurate staging, prognosis, and treatment planning, especially when imaging results are inconclusive or when marrow involvement is suspected despite a negative scan. In the study by Li et al. (31), the authors described false-negative PET/CT scan for bone marrow involvement in 31% of their study patients, which further emphasizes the importance of histological confirmation in the bone marrow.

Thus, pathologists should be aware of the patterns of bone marrow involvement in CHL and should be particularly cautious when encountering biopsies showing increased reticulin fibrosis with alternating cellularity or a histiocyte-rich background. In these cases, particular attention to finding RS cells and immunophenotypic confirmation are extremely important to avoid misdiagnosis.

## Discussion and conclusion

Bone marrow evaluation is critical for assessing hematologic disorders, including lymphomas, leukemias, myeloproliferative diseases, and myelodysplastic syndromes. However, bone marrow biopsy (BMB) and aspirate (BMA) can present several pitfalls that may lead to misinterpretation or missed diagnoses. These pitfalls can arise due to sampling issues, technical challenges, or inherent characteristics of the disease itself. In a study of 130 cases (30 excluded), BMB proved more reliable in conditions with marrow fibrosis-like myelofibrosis (MF), where BMA often failed due to dry taps (35). The bone marrow is heterogeneous, and focal areas of involvement, specifically in lymphomas, can be missed due to sampling errors (36). While BMA showed high sensitivity for diagnosing nutritional anemia (100%) and acute leukemia (100%), BMB was superior for diagnosing hypoplastic/aplastic anemia, MF, and granulomatous inflammation (35, 37). BMB also provided critical prognostic information on chronic leukemias, multiple myeloma (MM), and lymphomas where ancillary studies like immunohistochemistry can be performed (38). Similarly, inadequate aspirates due to “dry tap” and hemodilution can result in insufficient cellular material for accurate diagnosis, while a biopsy can provide architectural information but may not contain enough marrow elements (39, 40). In a study of 2,235 concurrent BMA and BMB examination, 3.9% were “dry taps.” Of these, the majority were diagnosed with a pathological condition like marrow fibrosis or hypercellularity (e.g., metastatic carcinoma, CML, idiopathic myelofibrosis, hairy cell leukemia), and only a small percentage of cases (6.9%) were normal (39). In a study by Goyal et al. (6), BMA demonstrated high sensitivity for acute leukemia (89.4%) and multiple myeloma (88.5%), moderate sensitivity for non-Hodgkin lymphoma (67.6%) and non-hematopoietic metastases (58.3%), and low sensitivity for aplastic anemia (38.5%) and Hodgkin lymphoma (5%) (6). Aspirate was not useful in cases of granulomatous myelitis and myelofibrosis in that study. Furthermore, lymphoma detection rates increased with trephine biopsy length, with the highest positivity (68.9%) observed in the 17–20-mm group, and no additional benefit was noted beyond 20 mm (41). Furthermore, decalcification of core biopsies, particularly with strong acids such as hydrochloric acid, can degrade antigenicity and result in poor immunohistochemical staining or even false-negative results, thereby complicating diagnosis. Studies have consistently shown that such acid-based methods negatively impact both protein integrity and immunohistochemical staining (IHC) performance (42) and can lead to improper staining or even negative staining in certain cases, causing diagnostic difficulties (43, 44). It is recommended that gentler agents like EDTA better preserve antigenic epitopes and are therefore preferable when accurate immunophenotyping is required (44). Finally, the architecture and patterns of involvement of the marrow may look different than the lymph node, in cases of lymphomas secondarily involving the marrow, which can lead to potential pitfalls of misdiagnosis (45, 46). Bone marrow involvement in CHL is uncommon, and subtyping based on marrow specimens is discouraged due to possible histopathologic discordance with the primary tumor (31). In the study by Li et al. (31), among the 23 cases studied, mixed cellularity (MC) and nodular sclerosis (NS) were the predominant subtypes. Two patterns of marrow involvement were observed: pattern A (fibrous): space-occupying lesions with alternating hypo- and hypercellular areas, fibrotic stroma, and dilated sinusoids; and pattern B (histiocyte-rich): ill-defined granuloma-like lesions where histiocytes blended with normal hematopoietic and inflammatory cells. Pattern A was significantly more frequent in the NS subtype and had less EBV positivity as compared to MC cases. Moreover, Hodgkin and Reed–Sternberg (HRS) cells were present in all cases, with lacunar variants predominantly seen in the NS subtype (31). Thus, with challenging cases, looking at morphology carefully and finding an HRS cell are helpful, and performing ancillary studies like immunohistochemistry might be the key to an accurate diagnosis. Please find **Table 1** describing the frequency of bone marrow involvement across lymphoma subtypes (47).

**Table 1:** Frequency of bone marrow involvement across lymphoma subtypes (47).

Lymphoma subtype	Approximate frequency of bone marrow involvement (%)	Typical pattern of infiltration
Chronic lymphocytic leukemia/small lymphocytic lymphoma (CLL/SLL)	80–90	Diffuse or interstitial
Mantle cell lymphoma (MCL)	55–75	Nodular or interstitial, occasionally paratrabecular
Follicular lymphoma (FL)	50–60	Paratrabecular
Marginal zone lymphoma (MZL)	40–50	Nodular, interstitial, or mixed
Diffuse large B-cell lymphoma (DLBCL)	10–20	Focal or diffuse, often discordant morphology
Lymphoplasmacytic lymphoma/Waldenström macroglobulinemia (LPL/WM)	70–90	Diffuse or interstitial
Hodgkin lymphoma (HL)	5–10	Focal, often missed on aspirate
Peripheral T-cell lymphoma, NOS (PTCL-NOS)	20–30	Focal or diffuse
Angioimmunoblastic T-cell lymphoma (AITL)	30–50	Diffuse, polymorphous infiltrate
Anaplastic large-cell lymphoma (ALCL)	10–20	Focal, often sinusoidal

Although pathologists should be aware of these marrow patterns and potential pitfalls, to optimize diagnostic accuracy and patient outcomes, hematologists should prioritize comprehensive and high-quality bone marrow sampling when evaluating hematologic disorders, particularly lymphomas and other malignancies with focal or variable marrow involvement (48). When possible, we need to ensure that both aspirate and core biopsy were obtained and that the biopsy length met recommended standards (≥1.5–2 cm evaluable length) (49). In cases of “dry tap” or hemodilution, prompt communication with pathology teams is crucial to consider repeat sampling or adjunct studies (e.g., flow cytometry on peripheral blood or imaging-guided biopsy) (40). Hematologists should also be aware of the limitations of marrow sampling in certain conditions, such as Hodgkin lymphoma, and interpret marrow findings in the broader context of clinical, radiologic, and nodal histopathology (45, 46). Finally, early integration of ancillary techniques—such as immunohistochemistry, molecular testing, and PET-CT correlation—should be considered in diagnostically challenging cases or when marrow findings are discordant with the clinical picture.

## Data Availability

The original contributions presented in the study are included in the article/supplementary material. Further inquiries can be directed to the corresponding author.
